# Feasibility and efficacy of the forced oscillation technique in patients with lysosomal storage diseases

**DOI:** 10.1038/s41598-025-92076-8

**Published:** 2025-02-28

**Authors:** Afaf Alblooshi, Nuha Al Zaabi, Ghaya Albadi, Fatma A. Al-Jasmi

**Affiliations:** 1 Department of Medical Education, College of Medicine and Health Sciences, United Arab Emirates University, Al Ain, United Arab Emirates; 2 Department of Pediatrics, Fujairah Hospital, Emirates Health Services, Fujairah, United Arab Emirates; 3 Department of Pediatrics, College of Medicine and Health Sciences, United Arab Emirates University, Al Ain, United Arab Emirates; 4 Department of Genetics and Genomics, College of Medicine and Health Sciences, United Arab Emirates University, Al Ain, United Arab Emirates; 5 College of Medicine and Health Sciences, United Arab Emirates University, P.O. Box 17666, Al Ain, United Arab Emirates

**Keywords:** Lysosomal storage diseases, Forced Oscillation technique, Lung diseases, Lung function, Metabolic, Clinical genetics, Medical genetics, Health care, Medical research

## Abstract

Lysosomal storage disorders (LSDs) are a diverse group of inborn errors of metabolism, which involves the storage of macromolecules within the lysosome due to the absence of or deficiency in lysosomal enzymes. In this condition, respiratory dysfunction is the common cause of morbidity and mortality. Thus, an objective measurement examination of pulmonary function, such as spirometry, is essential for the diagnosis and management of respiratory conditions. However, spirometry requires active patient involvement. Meanwhile, the forced oscillation technique (FOT) is performed during tidal breathing and is feasible in patients who cannot perform spirometry. Thus, the current study aimed to assess the feasibility and efficacy of FOT in assessing respiratory function among patients with LSD. This study was conducted at the outpatient department of Tawam Hospital, Al-Ain, the United Arab Emirates. Patients with confirmed LSD were included in the analysis. Information on respiratory history was obtained using a standardized respiratory questionnaire, and lung function was assessed using FOT. The main outcomes were respiratory resistance (Rrs5), reactance at 5 Hz (Xrs5), and area under the reactance curve (AX). In total, 35 patients with LSD were assessed. FOT was used to evaluate 16 (47%) patients. Of the patients who were successfully assessed using FOT, 10 presented with abnormal outcomes. Hence, there was an association between lung function assessed using FOT and spirometry results in these patients. FOT can help obtain lung function measurements in patients with LSD. In this study, the patients commonly presented with abnormal FOT outcomes.

**Supplementary Information:**

The online version contains supplementary material available at 10.1038/s41598-025-92076-8.

## Background

Lysosomal storage disorders (LSDs) are a diverse group of inborn errors of metabolism, which involves the storage of macromolecules within the lysosome due to a deficiency in one or more lysosomal enzymes or transporters. LSDs are multisystemic diseases with variable presentation. Based on the nature and storage site of the specific macromolecules, these conditions have a wide spectrum of manifestations.

Respiratory complications are one of the main causes of mortality and morbidity in patients with LSDs and, more specifically, in children with a severe form of the disease, such as mucopolysaccharidosis (MPS), mucolipidosis II/III, and Gaucher type II^1–3^. In some patients with LSD, such as MPS, Niemann–Pick disease, and Gaucher disease, pulmonary manifestations are among the initial presenting symptoms^2^. That is, some reports have shown that over 40% of patients with Gaucher and Niemann–Pick disease present with respiratory symptoms prior to diagnosis^4^. Therefore, to diagnose and manage underlying respiratory conditions in patients with LSD, lung function measures should be assessed.

Objective lung function tests are important for the diagnosis and management of respiratory conditions in children with LSD. Spirometry is the gold standard method for assessing lung function in older children and adults. However, to obtain acceptable and repeatable outcomes, this test requires effort and a high level of cooperation^5–7^. Thus, in patients who cannot perform spirometry, assessment is affected by the lack of objective measures used to evaluate lung function. The forced oscillation technique (FOT) is a potential lung function method that can be used in children who cannot perform spirometry^8–10^. FOT is applied during tidal breathing and is feasible in patients aged 2 years or older^11,12^. This method has been used for the assessment of respiratory function in both the clinical and research field. Hence, the current study aimed to assess the feasibility and efficacy of FOT in detecting respiratory involvement in patients with LSD.

## Methods

### Participants

This prospective case series study was conducted at Tawam Hospital, Al Ain, Abu Dhabi, between January 2016 and April 2019. Including patients diagnosed with LSD. Written informed consent was obtained from participants aged ≥18 years and from their parent if they were aged <18 years. Disease severity was assessed using international guidelines for LSDs, and genotyping was performed accordingly^13–16^.

Demographic data including height and weight, were obtained from all participants. Respiratory history was collected using a modified respiratory questionnaire^17^. This questionnaire was translated from English (original language) to Arabic and then back-translated into English by two experts in the field of respiratory who are proficient in both Arabic and English. This served as the validation method employed. The evaluation process aligns with the guidelines of the Global Asthma Network. All measurements were taken during a single visit.

### Spirometry

Lung function was evaluated using spirometry on the same day as the participants’ routine clinical care, but only if they reported respiratory symptoms, in accordance with the guidelines for each specific LSD. Spirometry was performed according to the ATS/ERS guidelines^18^.

### Forced Oscillation technique

Lung function was further assessed using FOT with a commercial device (tremoFlo™ C-100; tremoFlo™ software, version 1.0.43/Build 39; Thorasys Medical Systems, Montreal, QC, Canada). Pseudorandom signals at frequencies ranging from 5 to 37 Hz were applied, and the measurements were performed as per the international guidelines^8^ and previously published work on a healthy population using FOT^12^.

In cases where baseline FOT measurements were abnormal, the test was repeated 15 min after the administration of 400 mcg of salbutamol via a pressurized metered-dose inhaler (Ventolin, GlaxoSmithKline) and spacer device (Lite Aire, Thayer Medical). The FOT outcomes included:

- Mean respiratory resistance at 5 Hz (Rrs5, in cmH_2_O.s.L − 1),
- Mean respiratory reactance at 5 Hz (Xrs5, in cmH_2_O.s.L − 1), and.
- Area under the reactance curve (AX, cm/L).

All FOT outcomes were reported as z-scores using reference equations based on the participants height and age. Two reference equations were used:

- The first equation was applied to participants with height ≤ 156 cm, as described in a previous study^12^.
- The second equation was used to participants with a height ≥ 156 cm^19^.

Two reference equations were necessary due to the broad age range and height variability within the study population, as well as the absence of a single local reference equation suitable for the cohort.

## Bronchodilator responsiveness

Responsiveness to bronchodilators was defined as the percentage relative change from the baseline value for each FOT variable^9^. A clinically relevant BDR was defined as an absolute change in the following:

- 34% change in Rrs5,
- 50% change in Xrs5, and
- 81% change in AX^20^.

To interpret the z-scores, the lower limit of normal values was defined by the 5th percentile (− 1.64 z-scores) and the upper limit by the 95th percentile (+ 1.64 z-scores), as recommended by the American Thoracic Society and the European Respiratory Society^8^.

## Results

Table 1 compares the respiratory history of patients with successful and unsuccessful Forced Oscillation Technique (FOT) outcomes based on the severity of lysosomal storage diseases (LSDs), which are associated with varying degrees of respiratory comorbidities. A total of 35 patients diagnosed with LSDs, aged between 2 and 59 years, were enrolled in the study, with 43% being women. Of these, 26 patients (74%) had a history of respiratory symptoms that required emergency room visits within the last 12 months. Seventeen patients (49%) had a history of wheezing, and 18 patients (51%) were using respiratory medications (Table 1). This comparison aims to explore how disease severity and associated respiratory complications may impact the success of FOT in this population.

**Table 1:** Respiratory history in patients with successful and unsuccessful FOT.

Respiratory History	Patients with unsuccessful FOT (*n* = 19)	Patients with successful FOT (*n* = 16)
Age (years), mean ± SD (median) Range	10 ± 6 (10) 2–24	15 ± 13 (13) 3–59
History of wheezing	10 (53%)	7 (44%)
History of wheezing within the last 12 months	7 (37%)	4 (25%)
History of using respiratory medications	12 (63%)	6 (38%)
ER visits due to respiratory symptoms	15 (79%)	11 (69%)
History of pneumonia within the first year of life	11 (58%)	6 (38%)
History of URTI within the last 12 months	16 (84%)	10 (63%)
History of tonsillectomy and/or adenoidectomy	9 (47%)	2 (13%)
History of feeding difficulties	10 (53%)	1 (6%)
History of snoring	5 (26%)	8 (50%)
History of sleep apnoea	2 (11%)	2 (13%)
Family history of atopy	11 (58%)	6 (38%)

FOT: forced oscillation technique; ER: emergency room; URTI: upper respiratory tract infection

Lung function assessment using FOT was successful in 16 patients with LSD and unsuccessful in 19 patients. Detailed data on demographic characteristics, mutations, and clinical history for those with successful and unsuccessful FOT measurements are presented in the online supplementary material (Table 1A–S and 1B–S, respectively). In total, 19 patients with unsuccessful FOT could not perform spirometry due to intellectual disability (*n* = 15), tracheostomy (*n* = 3), and young age (*n* = 1). Meanwhile, of 16 patients with successful FOT measurements, only three performed spirometry, and the remaining patients did not conduct spirometry due to intellectual disability (*n* = 2), young age (*n* = 3), poor patient effort (*n* = 2), absence of respiratory symptoms (*n* = 4), and lack of interest even though the procedure was requested (*n* = 2) (Tables 2 and 3). Patients with unsuccessful FOT had more respiratory manifestations than those with successful FOT, as shown in Table 1. Approximately 63% of patients with unsuccessful FOT and 38% of those with successful FOT were taking respiratory medications.

**Table 2:** Lung function using the forced Oscillation technique (FOT) in patients with mucopolysaccharidosis.

ID	Gender, Age (y)	Disease classification	Mutation	Metabolic treatment	Respiratory treatment	Rrs5 z-scores		Relative change in Rrs5 (%)	Xrs5 z-scores		Relative change in Xrs5 (%)	AX z-scores		Relative change in AX (%)	FOT results	Spirometry	Sleep study
						Pre-BD	Post-BD		Pre-BD	Post-BD		Pre-BD	Post-BD				
1	F, 5	I	*IDUA*: c.1469T > C^a^, (p.L490P) NM_000203.3	Laronidase started at 1 year of age	-	2.46	1.97	9.31%	1.72	1.14	8.58%	2.23	1.90	11.93%	Abnormal baseline Rrs5, Xrs5, and AX without clinically significant BDR	Not requested because the patient was young	OSA, improved after adenoidectomy
2	F, 6	I	*IDUA*: c.1861 C > T/c.1189 + 1^b^G > A/c.299 + 6 C > T, (p.Arg621*/intronic apparent splicing/intronic) NM_000203.3	HSCT at 1 year of age	-	1.17	-0.76	31.74%	2.27	2.26	0.15%	2.21	0.16	56.83%	Normal baseline Rrs5 with borderline BDR, abnormal baseline Xrs5 and AX without clinically significant BDR	Not requested because the patient was young	-
16	F, 17	IVA	-	Elosulfase alfa started at 14 years of age	SABA	2.02	2.29	− 5.61%	2.16	3.84	-23.64%	3.92	3.65	7.06%	Abnormal baseline Rrs5, Xrs5, and AX without clinically significant BDR	Not successful due to poor patient effort	Mild OSA
17	F, 15	IVA	-	Elosulfase alfa started at 13 years of age	-	1.26	-0.36	27.50%	4.74	1.21	38.27%	2.42	1.30	36.97%	Normal baseline Rrs5 with borderline BDR. Abnormal baseline Xrs5 and AX without clinically significant BDR	Not successful due to poor patient effort	Normal
18	M, 9	IVA	*GALNS*: c.319 G > A^a^, (p.Ala107Thr) NM_000512	Elosulfase alfa started at 5 years of age	SABA, ICS discontinued once ERT has been started	0.05	-	-	-1.67	-	-	1.64	-	-	Normal baseline Rrs5, Xrs5, and AX	Requested by the physician but patient did not perform the procedure	-
19	M, 10	VI	*ARSB*: c.944G > A, (p.Arg315Gln) NM_000046	Galsulfase started at 3 years of age	SABA	4.95	2.96	32.50%	10.87	-0.18	72.49%	7.59	3.75	59.18%	Abnormal baseline Rrs5, Xrs5, and AX with clinically significant BDR in Rrs5 and Xrs5	Requested by the physician but the patient did not perform the procedure	Severe OSA

Rrs5: respiratory resistance at 5 Hz; Xrs5: respiratory reactance at 5 Hz; AX: area under the reactance curve; HSCT: hematopoietic stem cell transplantation; SABA: short-acting β-agonists; LABA: long-acting β-agonists; ICS: inhaled corticosteroids; OSA: obstructive sleep apnea; ERT: enzyme replacement therapy.

a homozygous mutation

b Novel variant

**Table 3:** Lung function using the forced Oscillation technique (FOT) in patients with lysosomal storage diseases (LSD).

ID	Gender, age (years)	Disease	Mutation reference sequence	Disease severity score	Metabolic treatment	Respiratory treatment	Rrs5 z-scores		Relative change in Rrs5 (%)	Xrs5 z-scores		Relative change in Xrs5 (%)	AX z-scores		Relative change in AX (%)	FOT results	Spirometry
							Pre-BD	Post-BD		Pre-BD	Post-BD		Pre-BD	Post-BD			
21	M, 59	Fabry	*GLA*: c.265 C > T^b^ (p.Leu89Phe) NM_000169.2	38/80^a^ (PNS:3, renal: 30, cardiac 2, CNS 2, patient reported 1)	Agalsidase beta	-	0.02	-	-	0.68	-	-	-	-	-	Normal baseline Rrs5 and Xrs5	Not performed because the patient did not have respiratory symptoms
22	M, 28	Fabry	*GLA*:c.1277_1278delAA, (p.K426fs) NM_000169.2	32/80^a^ (PNS:0, renal: 30, cardiac 0, CNS 0, patient reported 2*)*	Agalsidase beta	-	0.47	-	-	1.23	-	-	-	-	-	Normal baseline Rrs5 and Xrs5	Not performed because the patient did not have respiratory symptoms
23	M,12	Gaucher type I*	*GBA*: c.854T > C^a^, (p.Phe285Ser) CCDS 1102.1	10/19^b^	Velaglucerase alfa	SABA, ICS anti-cholinergic,	2.19	− 0.13	36.80%	1.51	-0.45	40.05%	2.63	0.46	74.83%	Abnormal baseline Rrs5 with significant BDR, normal baseline Xrs without significant BDR, abnormal baseline AX with borderline BDR	With mild obstruction with positive bronchodilator response
24	F, 17	Gaucher type I	*GBA*: c.1397T > G/c.1448T > C, (p.Ile466Ser/p.Leu483Pro) NM_000157.3	6/19^b^	Velaglucerase alfa	-	-0.02	-	-	1.08	-	-	-	-	-	Normal baseline Rrs5 and Xrs5	Normal
35	M, 3	Gaucher type I#	c.1448T > C^a^ (p.Leu483Pro), NM_000157.3	4/19^b^	Imiglucerase	-	-0.10	-	-	0.70	-	-	1.75	-	-	Normal baseline Rrs5, Xrs5 and abnormal AX	Not feasible because of the patients’ age
25	M, 13	Niemann–Pick type C	*NPC1*: c.1408G > C^a^/c.2509 A > G^a^, (p.Ala470Pro/p.Ile837Val) NM_000271	6/18^c^	Miglustat	-	0.07	-	-	0.64	-	-	-	-	-	Normal baseline Rrs5 and Xrs5	Failed spirometry as the patient had progressive intellectual disability
26	F, 14	Niemann–Pick type C	*NPC1*: c.2130G > C^b^/c.2660 C > T, (p.Gln710His/p.Pro887Leu) NM_000271.4	10/18^c^	-	-	3.87	1.49	37.62%	5.17	1.47	49.12%	4.88	1.73	77.26%	Abnormal baseline Rrs5 with significant BDR, abnormal baseline Xrs5 and AX with borderline BDR	Failed spirometry as the patient had significant progressive intellectual disability
27	M, 19	Saposin deficiency	*PSAP*: c.1005 + 1G > A^a^ CCDS7311.1	-	-	-	-0.14	-	-	0.55	-	-	-	-	-	Normal baseline Rrs5 and Xrs5	Not requested because the patient did not have respiratory symptoms
28	M, 7	Saposin deficiency	*PSAP*: c.1005 + 1G > A^a^ CCDS7311.1	-	-	-	-0.05	-	-	0.79	-	-	0.27	-	-	Normal baseline Rrs5, Xrs5 and AX	Not requested because the patient did not present with respiratory symptoms
30	M, 12	Mucolipidosis III	*GNPTG*: c.499dupC^a^, (p.Leu167Profs*32) NM_032520.3	-	-	SABA, montelukast	2.13	-0.25	37.65%	0.55	-0.04	12.81%	1.51	0.09	60.67%	Abnormal baseline Rrs5 with significant BDR, normal Xrs and AX without significant BDR	With mild airway obstruction with positive bronchodilator response

^a^, Fabry Disease Severity Scoring System (DS3) Giannini et al., 2010; ^b^, Gaucher Disease Type 1 Severity Scoring System (GD-DS3) Weinreb et al., 2010; ^c^, NP-C functional disability scale Iturriaga et al., 2006, * this patient has atypical GD1 he has ichthyosis and vocal cord nodules but no neurological manifestation, # this patient has neuropathic mutation but clinically he has no neurological manifestation yet.

BD: bronchodilator; BDR: bronchodilator response; PNS: peripheral nervous system; CNS: central nervous system; SABA: short-acting β2-agonists; ICS: inhaled corticosteroids.

Five of six patients with MPS who had a successful FOT had impaired baseline lung function, as represented by either Rrs5, Xrs5, or AX z-score (Table 2). Of patients with successful FOT, five were on enzyme replacement therapy, and one received bone marrow transplantation (BMT). Four patients underwent sleep study. Of these patients, one had normal results, two had mild obstructive sleep apnea, and one had severe obstructive sleep apnea. All six patients with MPS did not perform spirometry due to varying reasons (Table 2).

Two patients with Fabry disease who had successful FOT had normal baseline lung function as represented by Rrs5, Xrs5, and AX z-scores. In these patients, the assessment of lung function using spirometry was not performed, as per the guidelines, because they did not have respiratory symptoms^21^.

Of three patients with Gaucher type I, one with a severe form of the disease (ID 23, severity score: 10/19) had an abnormal baseline lung function as represented by Rrs5 and AX z-scores with positive BDR in Rrs5 only. Moreover, the patient had abnormal spirometry results with mild obstruction and positive BDR. The other two patients (ID 24 and 35) with a mild form of the disease (severity score: 6/19 and 4/19, respectively) had normal Rrs5 and Xrs5 z-scores, and the patient with ID 35 had an abnormal AX z-score. The older patient (ID 24) had normal spirometry results. However, the test was not feasible in the younger patient (ID 35) (Table 3).

Of two patients with Niemann–Pick type C, one with advanced disease (ID 26) who is on supportive therapy (severity score: 10/18) had an abnormal baseline lung function as represented by Rrs5, Xrs5, and AX z-scores with significant BDR in Rrs5 and borderline BDR in Xrs5 and AX. Meanwhile, the second patient (ID 25) who is on substrate reduction therapy (severity score: 6/18) had normal baseline Rrs5 and Xrs5 z-scores. Spirometry was not successful in both patients with Niemann–Pick type C due to lack of cooperation (Table 3).

The siblings with saposin deficiency had normal baseline Rrs5, Xrs5, and AX z-scores. Spirometry was not performed in these patients because they did not have any history of respiratory symptoms. The patient with mucolipidosis III had an abnormal baseline Rrs5 z-scores with significant BDR and normal baseline Xrs5 and AX z-scores without significant BDR. Spirometry revealed mild obstruction with positive bronchodilator response (Table 3).

## Discussion

Respiratory complications are one of the main causes of mortality and morbidity in patients with LSD^1–3^. Conventional respiratory function tests, including spirometry, are difficult to perform in most patients with LSD due to intellectual disability and respiratory manifestations, which commonly start at a young age.

In this study, lung function assessment using FOT was not feasible in 54% of participants due to intellectual disability and lack of cooperation. That is, all patients with Sanfilippo disease (MPSIII) were not assessed using FOT. Patients with unsuccessful FOT had more respiratory symptoms than those with unsuccessful FOT. Patients with more respiratory symptoms have advanced disease with significant intellectual disability inhibiting the performance of FOT.

Lung function assessment using FOT was feasible in 46% (*n* = 16) of participants (Tables 2 and 3). Of these patients, eight (50%) exhibited abnormal baseline lung function based on the assessment using FOT, and four with abnormal baseline lung function had clinically significant responses to bronchodilators. Of 16 patients with successful FOT, 6 did not perform spirometry due to young age (< 6 years, *n* = 3) and poor patient effort (*n* = 3). FOT was successful in this group because it requires passive cooperation, unlike spirometry that requires active cooperation. This study showed that FOT may be suitable in patients with LSD who cannot perform spirometry, as measurements are performed during tidal breathing and forced expiratory maneuvers are not required.

### Correlation between FOT and respiratory symptoms in patients with LSD

Most patients with LSD had a history of respiratory symptoms, which is consistent with the clinical characteristic of these disorders. Patients with LSD have respiratory manifestations that originate from airway abnormalities and alterations in respiratory mechanics^2,22–24^. In patients with LSD, respiratory manifestations vary according to individual diseases and its severity.

### Use of FOT in patients with MPS

Mucopolysaccharidoses (MPSs) are inherited progressive multisystemic metabolic diseases associated with deficiencies in enzymes involved in the degradation of glycosaminoglycans (GAGs)^25^. MPS patients may have significant pulmonary disease due to multiple abnormalities manifesting as reduction in vital capacity (VC)^26^. Lower airway involvement may be caused by the accumulation of GAGs in the airspaces and interstitium or chronic airway aspiration due to swallowing difficulties associated with neuromotor delay^2^. In addition to diaphragm compression caused by hepatosplenomegaly^27–29^, pulmonary function can also be affected by short stature and skeletal dysplasia^2^. The combined effect of these alterations in respiratory mechanics coupled with airway abnormalities might lead to significant respiratory manifestations in these patients.

In our study, FOT was feasible in two patients with MPS I (100%). Despite early intervention with enzyme replacement therapy (ERT) and hematopoietic stem cell transplantation (HSCT), both patients had abnormal baseline pulmonary function based on an assessment using FOT, which did not improve significantly after the administration of bronchodilator. Therefore, the use of FOT in these patients detected early respiratory involvement, and spirometry was not feasible in both patients due to young age (Table 2).

In three patients with MPS IVA, FOT was successful (100%), and all patients had abnormal FOT outcomes. However, the result was worse in two older patients with MPS IVA who initially received ERT at an older age compared with the younger patient who received ERT at 5 years of age (Table 2). The use of FOT in these patients detected respiratory involvement. However, spirometry was not feasible in two older patients due to poor patient effort. This finding is consistent with the progressive nature of MPS IVA and better outcomes with the early initiation of treatment^30^.

In two patients with MPS VI, FOT was feasible in only one patient as the other one had tracheostomy. The patient with successful FOT had significant pulmonary involvement, as represented by abnormal baseline Rrs5, Xrs5, and AX z-scores with significant BDR response in Xrs after the administration of bronchodilators (Table 2; ID 19). This may be explained by the presence of respiratory abnormalities, which were reported in the literature. These abnormalities were caused by skeletal, thoracic abnormalities and impairment of chest wall mechanics. Moreover, they were observed in addition to the accumulation of GAG throughout the tracheobronchial tree and infiltration of upper airway structures, which may lead to severe upper airway obstruction^2^. Interestingly, most MPS patients significantly responded to bronchodilators. Hence, the question on whether the use of respiratory medication can improve respiratory function in these patients has been raised.

### Use of FOT in patients with Fabry disease

Fabry disease is an X-linked LSD caused by deficiency in alpha-galactosidase A. This condition is characterized by the storage of neutral glycosphingolipids in almost all tissues and cells. Moreover, it may cause dysfunction in the affected cell, thereby triggering inflammation and/or fibrosis. Although pulmonary involvement in Fabry disease has not been confirmed, it may involve obstructive lung disease and interstitial lung disease in the later stages of the disease^22^. In our study, FOT was feasible in all patients with Fabry disease (*n* = 2). Moreover, the baseline lung function was normal in both patients using FOT.

### Use of FOT in patients with gaucher disease

Gaucher disease is the most prevalent LSD and is caused by the impaired activity of glucocerebrosidase. This phenomenon results in the accumulation of sphingolipid glucosylceramide in the spleen, liver, lungs, brain, and bone marrow^31^. Pulmonary manifestation is common, with varying severity, in patients with Gaucher disease, and a comprehensive evaluation of pulmonary involvement is recommended even in the absence of clinical symptoms^32^.

In our study, the use of FOT was feasible in three patients with Gaucher disease. The patient (ID 23) with a higher severity score (10/19) had an abnormal pulmonary function test based on the assessment using FOT as represented by abnormal Rrs5 and AX z-scores with significant BDR in Rrs5 and borderline response in Xrs5 and AX. The FOT result was correlated with both severity scores and spirometry finding, which showed airway obstruction with positive BDR (Table 3). Moreover, this patient (ID 23) had interstitial lung disease, which was observed on high-resolution computed tomography (HRCT) scan of the chest. Moreover, nodules and septal thickening consistent with interstitial lung disease were observed. Of two patients with a mild form of Gaucher disease (severity score: 6/19 and 4/19), one had normal FOT and spirometry results (ID 24). However, the younger patient (ID 35) had normal Rrs5 and Xrs5 z-scores and abnormal AX z-score, and spirometry was not feasible in this patient due to young age (3 years). Moreover, the patient had the L483P variant, known previously as L444P, which might be correlated with pulmonary involvement. Accordingly, in this study, the use of FOT in patients with Gaucher disease revealed impaired respiratory function in patients with the novel F285S variant, indicating an underlying respiratory involvement, which was not reported previously.

### Use of FOT in patients with Niemann–Pick type C

Niemann–Pick disease type C is a complex lipid storage disorder caused by defects in cholesterol trafficking, mainly affecting the neurological system. The coexistence of Niemann–Pick disease type C with pulmonary alveolar proteinosis, which contributes to mortality, has been presented in several studies. In our study, the use of FOT was feasible in assessing lung involvement in two patients with Niemann–Pick disease type C. These patients had different severity scores. That is, one (ID 26) had a severe form of the disease (on supportive therapy with a severity score of 10/18), and the other one (ID 25) had a mild form of the disease (severity score: 6/18). The use of FOT revealed respiratory involvement in patients with the advanced type of Niemann–Pick type C. These patients had impaired lung function as represented by baseline Rrs5, Xrs5, and AX z-scores with significant BRD in Xrs5 and borderline BDR in Xrs5 and AX. The second patient (ID 25) with a milder form of the disease who was managed with substrate reduction therapy had normal Rrs5, Xrs5, and AX z-scores. The use of spirometry in both patients with Niemann–Pick disease type C was not feasible due to lack of cooperation and progressive intellectual disabilities (Table 3). Therefore, the use of FOT in patients with Niemann–Pick disease type C revealed impaired respiratory function, which cannot be identified using spirometry.

### Limitation

The current study had several limitations. That is, it was not designed to evaluate the correlation between lung function assessed using FOT and the findings of spirometry. However, the test was requested for patients with respiratory symptoms. Of 16 patients with successful FOT, only 3 performed spirometry.

The second limitation is the use of two reference equations to calculate the reference value for each participant. These equations, as highlighted in the Method section, were utilized due to the wide age group and height of the participants^12,19^. Our previous study showed that the polish reference equation used is more suitable to our population than other reference equations^12^. Another limitation is the application of the reference equations on mixed population (which included both Emiratis and non-Emiratis) even though most patients were Emiratis (Table 1A-S and 1B-S). However, the non-Emiratis were from the population without an established reference equation. Therefore, to the best of our knowledge, the application of our locally derived reference value might be more appropriate due to the similar geographical and ethnic background between the assessed population and our local population.

## Conclusion

The findings of this study strongly suggest that lysosomal storage diseases (LSDs) play a significant role in respiratory abnormalities, although asthma and other factors may also contribute. The Forced Oscillation Technique (FOT) emerges as a valuable tool in this context, providing critical information for the early detection and practical follow-up of disease progression. FOT can also be used to evaluate the efficacy of enzyme replacement therapy, hematopoietic stem cell transplantation, substrate reduction therapy, and bronchodilators in patients with LSD. Hence, the regular use of FOT in these patients should be considered as part of routine clinical care.

To further delineate the specific contributions of LSDs and other potential influences, future research should adopt a multifactorial approach. Advanced imaging techniques, such as high-resolution computed tomography (HRCT) and magnetic resonance imaging (MRI), can be utilized to better characterize the structural and functional changes associated with respiratory involvement in LSDs. Additionally, longitudinal studies are recommended to track disease progression and assess the impact of therapeutic interventions on respiratory outcomes. Collaborative efforts between clinicians, radiologists, and researchers will be vital to advancing knowledge and improving patient care.

## Electronic supplementary material

Below is the link to the electronic supplementary material.

Supplementary Material 1

Supplementary Material 2

## Data Availability

The datasets used and/or analysed during the current study are available from the corresponding author on reasonable request.

## Abbreviations

AX: Area under the Reactance Curve
ATS/ERS: American Thoracic Society / European Respiratory Society
BDR: Bronchodilator Response
BMT: Bone Marrow Transplantation
CNS: Central Nervous System
CMHS: College of Medicine and Health Sciences
ERT: Enzyme Replacement Therapy
FOT: Forced Oscillation Technique
GAGs: Glycosaminoglycans
GD-DS3: Gaucher Disease Severity Scoring System
HRCT: High-Resolution Computed Tomography
HSCT: Hematopoietic Stem Cell Transplantation
ICS: Inhaled Corticosteroids
LABA: Long-Acting β-Agonists
LSD: Lysosomal Storage Diseases
MPS: Mucopolysaccharidosis
MRI: Magnetic Resonance Imaging
OSA: Obstructive Sleep Apnea
PNS: Peripheral Nervous System
Rrs5: Respiratory Resistance at 5 Hz
SABA: Short-Acting β-Agonists
URTI: Upper Respiratory Tract Infection
VC: Vital Capacity
Xrs5: Respiratory Reactance at 5 Hz
