# Supplementary material for: Feasibility and efficacy of the forced oscillation technique in patients with lysosomal storage diseases
Source: Sci Rep. 2025 Feb 28;15:7245. doi: 10.1038/s41598-025-92076-8 (PMC11871331; doi:10.1038/s41598-025-92076-8)
Supplement: Supplementary file 1 — Supplementary Material 1 [file 41598_2025_92076_MOESM1_ESM.docx]

| Table 1A-S. Demographics and questionnaire data of patients with successful FOT (n=16) | | | | | | | | | | | | | | | | | | | |
| --- | --- | --- | --- | --- | --- | --- | --- | --- | --- | --- | --- | --- | --- | --- | --- | --- | --- | --- | --- |
| *ID* | *Gender, Age(y)* | *Height in cm, (percentile)* | *Weight in Kg, (percentile)* | *BMI percentile* | *Disease* | *Mutation*  *Reference sequence* | *Ethnicity* | *History of wheeze ever* | *Age wheeze started (y)* | *History of wheeze in the last year* | *Respiratory medications* | *ER visit with respiratory problems in the last year* | *History of pneumonia in the first year of life* | History of common cold in the last year | History of Tonsillectomy and/or Adenoidectomy | History of feeding difficulties | History of Snoring | History of Sleep Apnoea | History of Atopy in the Family |
| 1 | F, 5 | 106, (22^rd^) | 20, (68^h^) | 92^th^ | MPS I | *IDUA*: c.1469T>C^a^, (p.L490P)  NM_000203.3 | Pakistan | Yes | 3 | Yes | - | No | Yes | No | No | No | No | No | No |
| 2 | F, 6 | 95, (0.0^th^) | 13, (0^th^) | 36^th^ | MPS I | *IDUA*: c.1861C>T/c.1189+1^b^G>A/c.299+6C>T,  (p.Arg621*/Intronic apparent splicing/Intronic)  NM_000203.3 | UAE | No | - | No | - | Yes | Yes | Yes | No | No | Yes | No | No |
| 16 | F, 17 | 102, (0^th^) | 27, (0^th^) | 86^th^ | MPS IVA | - | Somalia | No | - | No | SABA | No | No | No | No | No | Yes | No | No |
| 17 | F, 15 | 111, (0^th^) | 30, (0^th^) | 87^th^ | MPS IVA | - | Somalia | No | - | No | - | Yes | No | No | No | No | No | No | Yes |
| 18 | M, 9 | 90, (0^th^) | 15, (0^th^) | 82^nd^ | MPS IVA | *GALNS:* c.319 G>A^a^, (p.Ala107Thr)  NM_000512 | UAE | Yes | 3 | No | SABA, ICS | No | No | Yes | No | No | No | No | Yes |
| 19 | M, 10 | 110, (0^th^) | 24, (2^nd^) | 83^rd^ | MPS VI | *ARSB:* c.944G>A, (p.Arg315Gln)  NM_000046 | Sudan | Yes | 1 | Yes | SABA | Yes | Yes | Yes | Yes | No | Yes | Yes | No |
| 21 | M, 58 | 165, (5^th^) | 81, (77^th^) | 96^th^ | Fabry | *GLA:* c.265C>T^b^ (p.Leu89Phe)  NM_000169.2 | UAE | No | - | No | - | Yes | No | Yes | No | No | No | No | No |
| 22 | M, 28 | 164, (5^th^) | 55, (5^th^) | 28^th^ | Fabry | *GLA:*c.1277_1278delAA, (p.K426fs)  NM_000169.2 | UAE | No | - | No | - | Yes | No | No | No | No | No | No | No |
| 23 | M,12 | 138, (10^th^) | 31, (10^th^) | 30^th^ | Gaucher type I | *GBA:* c.854T>C^a^, (p.Phe285Ser)  CCDS 1102.1 | Palestine | Yes | 3 | Yes | SABA, Anti-cholinergic, ICS | No | No | Yes | No | No | No | No | Yes |
| 24 | F, 17 | 163, (45^th^) | 57, (59^th^) | 67^th^ | Gaucher type I | *GBA:* c.1397T>G/c.1448T>C, (p.Ile466Ser/p.Leu483Pro)  NM_000157.3 | Kazakhstan | No | - | No | - | Yes | No | No | No | No | No | No | No |
| 35 | M, 3 | 87, (14^th^) | 14, (61^st^) | 0^th^ | Gaucher type I | c.1448T>C^a^ (p.Leu483Pro),  NM_000157.3 | Syrian | Yes | 1 | Yes | - | Yes | No | Yes | No | No | Yes | Yes | Yes |
| 25 | M, 13 | 162, (61^th^) | 46, (48^th^) | 28^th^ | Niemann–Pick type C | *NPC1*: c.1408G>C^a^/c.2509A>G^a^, (p.Ala470Pro/p.Ile837Val)  NM_000271 | India | No | - | No | - | Yes | Yes | Yes | No | No | Yes | No | No |
| 26 | F, 14 | 150, (9^th^) | 35, (3^rd^) | 5^th^ | Niemann–Pick type C | *NPC1:* c.2130G>C^b^/c.2660C>T, (p.Gln710His/p.Pro887Leu)  NM_000271.4 | India | Yes | 1 | No | - | Yes | Yes | No | No | Yes | No | No | Yes |
| 27 | M, 19 | 163, (3^rd^) | 46, (0^th^) | 0^th^ | Saposin deficiency | *PSAP*: c.1005+1G>A^a^  CCDS7311.1 | UAE | No | - | No | - | Yes | No | Yes | Yes | No | Yes | No | No |
| 28 | M, 7 | 105, (0.3^th^) | 16, (0^th^) | 15^th^ | Saposin deficiency | *PSAP*: c.1005+1G>A^a^  CCDS7311.1 | UAE | No | - | No | - | No | No | Yes | No | No | Yes | No | No |
| 30 | M, 12 | 126, (0.3^th^) | 25, (1^st^) | 20^th^ | Mucolipidosis III | *GNPTG:* c.499dupC^a^, (p.Leu167Profs*32)  NM_032520.3 | UAE | Yes | 6 | No | SABA, Montelukast | Yes | Yes | Yes | No | No | Yes | No | Yes |
| ER; emergency room; F: Female; M: Male; MPS: Mucopolysaccharidoses; SABA: Short acting ß2 agonist; LABA: long acting ß2 agonist.  a homozygous mutation  b Novel variant | | | | | | | | | | | | | | | | | | | |
