# Supplementary material for: Feasibility and efficacy of the forced oscillation technique in patients with lysosomal storage diseases
Source: Sci Rep. 2025 Feb 28;15:7245. doi: 10.1038/s41598-025-92076-8 (PMC11871331; doi:10.1038/s41598-025-92076-8)
Supplement: Supplementary file 2 — Supplementary Material 2 [file 41598_2025_92076_MOESM2_ESM.docx]

| Table 1B-S. Demographics and questionnaire data of patients with unsuccessful FOT (n=19) | | | | | | | | | | | | | | | | | | | |
| --- | --- | --- | --- | --- | --- | --- | --- | --- | --- | --- | --- | --- | --- | --- | --- | --- | --- | --- | --- |
| *ID* | *Gender, Age(y)* | *Height in cm, (percentile)* | *Weight in Kg, (percentile)* | *BMI percentile* | *Disease* | *Mutation*  *Reference sequence* | *Ethnicity* | *History of wheeze ever* | *Age wheeze started (y)* | *History of wheeze in the last year* | *Respiratory medications* | *ER visit with respiratory problems in the last year* | *History of pneumonia in the first year of life* | History of common cold in the last year | History of Tonsillectomy and/or Adenoidectomy | History of feeding difficulties | History of Snoring | History of Sleep Apnoea | History of Atopy in the Family |
| 3 | M, 9 | 112, (0.0^th^) | 28, (18^th^) | 96^th^ | MPS II | *IDS:* c.1418delC, (p.Pro473LeufsX10) | Palestine | Yes | 10 | No | SABA, ICS, LABA | Yes | No | Yes | Yes | Yes | Yes | No | No |
| 4 | M, 10 | 129, (37^th^)  at age 8 | 27, (48^th^) at age 8 | 55^th^ | MPS IIIA | *SGSH:* c.672C>A ^a,b^, (p.Tyr224*)  NM_000199.3 | Egypt | No | - | No | SABA, ICS, LABA | Yes | No | No | No | Yes | No | No | No |
| 5 | M, 2 | 85, (10^th^) | 14, (76^th^) | 96^th^ | MPS IIIA | *SGSH:* c.672C>A ^a,b^, (p.Tyr224*)  NM_000199.3 | Syria | Yes | 1 | Yes | SABA | Yes | Yes | Yes | No | No | No | No | No |
| 6 | F, 10 | 123, (1^st^) | 26, (6^th^) | 44^th^ | MPS IIIA | *SGSH:* c.381delG ^a,b^, (p.Pro128Argfs*136)  NM_000199.3 | Sudan | No | - | No | - | No | No | Yes | Yes | Yes | No | No | No |
| 7 | M, 3 | 104, (98^th^) | 17, (90^th^) | 34^th^ | MPS IIIA | *SGSH*: c.381delG ^a,b^, (p.Pro128Argfs*136)  NM_000199.3 | Sudan | No | - | No | - | Yes | No | Yes | Yes | No | No | No | No |
| 8 | F, 11 | 133, (5^th^) | 31, (13^th^) | 51^st^ | MPS IIIB | *NAGLU:* c.1694G>T^a^, (p.R565L)  NM_000263.3 | UAE | Yes | 1 | Yes | SABA, ICS, LABA, Montelukast | Yes | Yes | Yes | No | Yes | Yes | Yes | Yes |
| 9 | M, 19 | 149, (0^th^) | 45, (0^th^) | 27^th^ | MPS IIIB | *NAGLU:* c.1694G>T^a^, (p.R565L)  NM_000263.3 | UAE | Yes | 2 | Yes | SABA, Anti-cholinergic | Yes | Yes | Yes | Yes | Yes | Yes | No | Yes |
| 10 | F, 12 | 137, (68^th^) at 9 years | 36, (82^nd^) at 9 years | 84^th^ | MPS IIIB | *NAGLU:* c.1694G>T^a^, (p.R565L)  NM_000263.3 | UAE | No | - | No | SABA | Yes | Yes | Yes | Yes | Yes | No | No | Yes |
| 11 | M, 9 | 118, (0.4^th^) | 28, (31^st^) | 90^th^ | MPS IIIB | *NAGLU:* c.1694G>T^a^, (p.R565L)  NM_000263.3 | UAE | Yes | 1 | Yes | SABA, ICS, Montelukast | No | Yes | Yes | Yes | Yes | Yes | Yes | No |
| 12 | M, 12 | 136, (0.6^th^) | 32, (3^rd^) | 31^st^ | MPS IIIC | *HGSNAT*: c.1600A>G^a,b^, (p.Asn534Asp)  NM_152419.2 | UAE | No | - | No | - | No | No | Yes | Yes | No | No | No | Yes |
| 13 | F, 12 | 146, (23^rd^) | 52, (80^th^) | 23^rd^ | MPS IIIC | *HGSNAT:* c.1348del^a^*,* (p.Asp450Ilefs*32)  NM_152419.2 | UAE | Yes | 3 | Yes | SABA | Yes | Yes | Yes | No | Yes | No | No | Yes |
| 14 | F, 6 | 118, (35^th^) | 26, (80^th^) | 91^st^ | MPS IIIC | *HGSNAT:* c.1348del^a^*,* (p.Asp450Ilefs*32)  NM_152419.2 | UAE | Yes | 2 | No | - | Yes | No | Yes | No | No | No | No | Yes |
| 15 | F, 4 | 104, (61^st^) | 19, (84^th^) | 92^nd^ | MPS IIIC | *HGSNAT*: c.1327G>A^a,b^, (p.Asp443Asn)  NM_152419.2 | UAE | No | - | No | - | Yes | No | Yes | No | No | No | No | Yes |
| 20 | F, 24 | 142, (0^th^) | 30, (0^th^) | 0^th^ | MPS VI | *ARSB:* c.979C>T*,* (p.Arg327*) | UAE | No | - | No | SABA, LABA | No | No | No | No | No | No | No | No |
| 29 | M, 6 | 108, (7^th^) | 15, (0^th^) | 0^th^ | Tay-Sachs | *HEXA:* c.2T>C^a^, (p.m1?)  NM_000520 | UAE | No | - | No | - | Yes | Yes | No | No | No | No | No | Yes |
| 31 | F, 10 | 107, (0^th^) | 19, (0^th^) | 42^th^ | Fucosidosis | *FUCA1*: c.558dupA^a^, (p.Glu187Argfs*10)  NM_000147.4 | UAE | Yes | 1 | Yes | SABA, ICS | Yes | Yes | Yes | Yes | Yes | No | No | Yes |
| 32 | F, 11 | 147, (65^th^) | 51, (92th) | 94^th^ | Alpha-Mannosidosis | *MAN2B1*: c.2356-2A>G^a,b^, (IVS192A>G)  NM_000528.3 | UAE | Yes | 1 | No | SABA | Yes | Yes | Yes | Yes | No | Yes | No | Yes |
| 34 | M, 3 | 89, (0^th^) | 15, (31^st^) | 0^th^ | Alpha-Mannosidosis | *MAN2B1*: c.2356-2A>G^a,b^, (IVS192A>G)  NM_000528.3 | UAE | Yes | 1 | Yes | SABA, ICS | Yes | Yes | Yes | No | Yes | No | No | Yes |
| 33 | M, 5 | 116, (61^st^) | 18, (19^th^) | 3^rd^ | Neuronal ceroid lipofuscinosis 6 | *CLN6:* c.407G>A^a^*,* (p.Arg136His)  NM_017882.2 | Egypt | No | - | No | - | Yes | Yes | Yes | No | No | No | No | No |
| ER; emergency room; F: Female; M: Male; MPS: Mucopolysaccharidoses; SABA: Short acting ß2 agonist; LABA: long acting ß2 agonist.  Patients with tracheostomy are highlighted  a homozygous mutation  b Novel variant | | | | | | | | | | | | | | | | | | | |
